# Osmolytes vs. Anabolic Reserves: Contrasting Gonadal Metabolomes in Two Sympatric Mediterranean Sea Urchins

**DOI:** 10.3390/metabo15120787

**Published:** 2025-12-10

**Authors:** Estela Carbonell-Garzón, Ricardo Ibanco-Cañete, Pablo Sanchez-Jerez, Frutos C. Marhuenda Egea

**Affiliations:** 1 Department of Marine Sciences and Applied Biology, University of Alicante, 03690 Alicante, Spain; estela.carbonell@ua.es (E.C.-G.); ricardo.ibanco@gmail.com (R.I.-C.); pablo.sanchez@ua.es (P.S.-J.); 2 Department of Biochemistry and Molecular Biology and Agricultural Chemistry and Edafology, University of Alicante, 03690 Alicante, Spain

**Keywords:** HR-MAS NMR spectroscopy, sea urchin metabolomics, *Paracentrotus lividus*, *Arbacia lixula*, gonadal biochemistry, osmolytes and amino acids, microbial interactions, reproductive physiology, ecological adaptation

## Abstract

**Background an Objectives:** The Mediterranean sea urchins *Paracentrotus lividus* and *Arbacia lixula* co-occur on shallow rocky reefs but display contrasting ecological and physiological traits. We compared their gonadal metabolomes to identify species-specific metabolic strategies. **Methods:** High-resolution magic angle spinning nuclear magnetic resonance (HR-MAS NMR) spectroscopy to intact gonadal tissues, combining multivariate chemometric modelling with targeted integration, boxplot-based univariate analysis and pathway analysis. **Results:** *A. lixula* showed an osmolyte- and redox-oriented phenotype with elevated betaine, taurine, sarcosine, trimethylamine (TMA), trimethylamine N-oxide (TMAO), carnitine, creatine, malonate, methylmalonate, uridine and xanthine. In contrast, *P. lividus* exhibited an amino-acid-enriched anabolic profile dominated by lysine, glycine and glutamine, together with higher levels of formaldehyde, methanol and 3-carboxypropyl-trimethylammonium. Pathway analysis indicated that *A. lixula* metabolites mapped onto glycine/serine–threonine metabolism and the folate-linked one-carbon pool, whereas *P. lividus* metabolites were enriched in glyoxylate/dicarboxylate, nitrogen and amino-acid pathways. These contrasting osmolyte–C_1_ versus nitrogen–amino-acid strategies are compatible with species-specific host–microbiota metabolic interactions inferred from published microbiome data. **Conclusions:** Overall, our results support a framework in which *A. lixula* adopts a resilience-oriented osmolyte strategy and *P. lividus* an efficiency-oriented anabolic strategy, highlighting HR-MAS NMR metabolomics as a powerful approach to investigate adaptive biochemical diversity in marine invertebrates.

## 1. Introduction

The Mediterranean Sea represents a unique natural laboratory where temperate and subtropical biota coexist under accelerating environmental change. Among the most influential benthic grazers shaping these coastal ecosystems are the sea urchins *Paracentrotus lividus* (the purple sea urchin) and *Arbacia lixula* (the black sea urchin). Both species are abundant on rocky reefs and play pivotal ecological roles as herbivores and bioengineers, modulating algal assemblages and promoting shifts between macroalgal forests and barren grounds [1,2]. Despite their spatial overlap, they exhibit contrasting ecological and physiological traits that underpin different adaptive strategies within the same environment.

*P. lividus* is a primarily herbivorous species with an optimal performance under moderate thermal regimes (15–20 °C), showing seasonal reproductive cycles synchronized with macroalgal availability [3,4,5]. Its metabolism is oriented toward the accumulation of amino acids and lipids, supporting a strategy of reproductive efficiency and energy storage [6]. In contrast, *A. lixula* is more thermotolerant and opportunistic, feeding on crustose algae, biofilms, and sessile invertebrates, and maintaining gonadal development even in nutrient-poor “barren” habitats [7,8,9]. Stable isotope and transcriptomic analyses have confirmed its omnivorous habits and enhanced capacity for oxidative stress resistance and immune plasticity [10,11]. These species therefore represent two alternative physiological solutions: one (in *A. lixula*) based on metabolic resilience and homeostatic control, and another (in *P. lividus*) based on anabolic efficiency and reproductive investment. Recent studies on scleractinian corals provide a clear illustration of this potential. Untargeted metabolomics and molecular networking applied to *Pocillopora meandrina* and *Seriatopora hystrix* have revealed species-specific enrichment of metabolites involved in energy metabolism, immune modulation, and redox homeostasis, tightly linked to their contrasting life-history strategies and environmental tolerance [12,13]. These coral studies demonstrate how metabolomic fingerprints can capture adaptive rewiring of biochemical networks in response to environmental drivers, a conceptual framework that we apply here to Mediterranean sea urchins.

Understanding the biochemical mechanisms underlying these divergent strategies is critical for predicting how Mediterranean ecosystems will respond to future environmental changes such as ocean warming and acidification. While previous research has examined sea urchin ecology, physiology, and genetics, the metabolic dimension of these adaptations remains poorly explored. Metabolomics—the comprehensive study of small-molecule metabolites—offers a powerful systems-level approach to link phenotype with physiology and environmental drivers [14,15]. It allows the detection of key metabolites involved in osmotic regulation, energy metabolism, and redox homeostasis, thereby revealing how organisms adjust their biochemical networks to changing conditions.

Among the analytical techniques available for metabolomics, high-resolution magic angle spinning nuclear magnetic resonance (HR-MAS NMR) spectroscopy provides distinct advantages for the study of marine invertebrates. HR-MAS enables the direct, non-destructive analysis of intact tissues, preserving the native biochemical environment and avoiding solvent extraction or derivatization [16]. This approach allows simultaneous identification of hydrophilic and hydrophobic metabolites, including amino acids, osmolytes, nucleotides, and lipids, while minimizing sample manipulation [17,18,19]. Moreover, HR-MAS NMR is highly reproducible and quantitative, making it ideal for comparative studies across species or environmental conditions. Its application in fish muscle, bivalves, and other marine taxa has yielded insights into nutritional status, stress responses, and environmental adaptation [19,20]; however, it has rarely been applied to echinoid gonadal tissues.

Sea urchin gonads are highly dynamic organs whose biochemical composition varies with sex, body size and gametogenic stage, as documented for *P. lividus* in different Atlantic and Mediterranean populations [3,4,5,6,21]. In the present work, we were not aiming to exhaustively characterize this intra-specific variability, but rather to obtain a controlled snapshot of species-level metabolic strategies. We therefore restricted our sampling to adult individuals collected from a single locality and season, interpreting the resulting metabolomic patterns as species-specific fingerprints expressed under shared spring environmental conditions.

In this context, the present study applies HR-MAS NMR-based metabolomics, combined with multivariate chemometric analyses (PLS-LDA and SPA), to compare the gonadal metabolic profiles of *Paracentrotus lividus* and *Arbacia lixula* collected from Mediterranean coastal habitats. Our objectives were to: (i) identify species-specific metabolic signatures reflecting physiological and ecological strategies; (ii) characterize the major classes of metabolites—amino acids, osmolytes, and secondary compounds—that differentiate the two species; and (iii) explore potential links between metabolomic traits, microbiota composition, and ecological adaptation.

We hypothesized that *A. lixula* and *P. lividus* exhibit distinct biochemical strategies reflecting their evolutionary divergence and trophic ecology: a resilience-oriented osmolyte metabolism in *A. lixula* versus an efficiency-oriented anabolic metabolism in *P. lividus*. By elucidating these metabolomic differences, this study contributes to a broader understanding of how closely related marine invertebrates achieve functional coexistence through divergent biochemical pathways.

## 2. Experimental Design and Procedure

### 2.1. Sample Collection

Adult individuals of *Paracentrotus lividus* and *Arbacia lixula* were collected in spring 2025 from shallow rocky reefs along the Western Mediterranean coast (38.3452° N, 0.4810° W). Sea urchins were hand-collected by free diving at depths ranging from 1 to 3 metres. Specimens were transported to the laboratory in insulated containers within two hours of collection to ensure tissue preservation.

Specimens of both species with macroscopically developed gonads were selected for HR-MAS analysis in order to minimize extreme differences in developmental stage. Sex and detailed gametogenic stage were not used as explicit stratification factors in the experimental design, and individual size was not included as a covariate in the multivariate models. Consequently, the metabolomic results presented here are interpreted as average species-level patterns for adult gonads under these shared spring field conditions.

### 2.2. Tissue Preparation

Upon arrival at the laboratory, the gonads were carefully dissected under sterile conditions. The tissues were rinsed with isotonic saline solution to remove any adherent debris and weighed to record wet mass. Gonadal samples were immediately snap-frozen in liquid nitrogen and stored at −80 °C until NMR analysis.

### 2.3. Chemicals

D2O (99.9%) and sodium (3-trimethylsilyl)-2,2,3,3-tetradeuteriopropionate (TSP) were purchased from Sigma–Aldrich (Sigma-Aldrich, St. Louis, MO, USA).

### 2.4. HR-MAS NMR Spectroscopy

Gonadal metabolomic profiling was performed using high-resolution magic angle spinning nuclear magnetic resonance (HR-MAS NMR) spectroscopy. Approximately 20 mg of frozen gonadal tissue was placed into a 4 mm zirconia rotor with 10 µL of phosphate-buffered solution in D_2_O (pH 7.4) containing 0.1% (*w/v*) TSP (trimethylsilylpropionate) as a chemical shift reference. Spectra were acquired at 500 MHz and 4 °C using a Bruker Avance III NMR spectrometer (Bruker, Rheinstetten, Germany), equipped with a HR-MAS probe. A Carr–Purcell–Meiboom–Gill (CPMG) pulse sequence was employed to suppress broad signals from macromolecules and enhance metabolite resolution.

For resonance assignment, additional 2D HR-MAS NMR experiments—including ^1^H–^1^H COSY-HR-MAS, ^1^H–^1^H TOCSY-HR-MAS and ^1^H–^13^C HSQC-MAS—were acquired on representative gonadal samples of both species. These 2D spectra, together with the corresponding 1D ^1^H HR-MAS data, were used to establish scalar and heteronuclear correlations and to identify the main resonances visible in the gonadal spectra, as summarized in Supplementary Table S1. All quantitative peak integrations and multivariate analyses in this study were performed exclusively on the 1D ^1^H HR-MAS spectra of sea urchin gonads. All spectra used in this work were acquired at the facilities of the Complutense University of Madrid (Centro de Bioimagen Complutense BIOIMAC) following the protocol outlined in the referenced article [18], using identical parameters for all experiments. 

The ^1^H-HR-MAS NMR technique employed in this study primarily facilitates qualitative comparisons among samples. Although it would provide reliable relative quantifications of certain metabolites, precise absolute quantifications were not within the scope of this analysis.

### 2.5. Data Processing and Statistical Analysis

NMR spectral data were processed using TopSpin 4.2.0 (Bruker, Rheinstetten, Germany) for baseline correction, phase adjustment, and metabolite identification. Signal alignment and normalization were performed prior to multivariate analysis.

Partial Least Squares–Linear Discriminant Analysis (PLS-LDA) was applied to the HR-MAS ^1^H-NMR spectral dataset to identify the resonances most responsible for discriminating between *Arbacia lixula* and *Paracentrotus lividus* gonads. This approach combines the dimensionality-reduction power of PLS with the classification capabilities of LDA, allowing robust modelling of high-dimensional, collinear metabolomic data. The libPLS toolbox provides integrated routines for pretreatment, cross-validation and PLS-LDA modelling; it is openly available at the project website https://www.libpls.net/ (accessed on 31 October 2025). The algorithm was implemented in MATLAB version 2024 (MathWorks, Natick, MA, USA), following the NIPALS procedure described by [22]. In this framework, the spectral data matrix *X* was decomposed as *X = T·P^′^* and the class vector *y* as *y = T·r^′^ = U·q^′^*, where *T* and *U* represent latent variables summarizing the systematic variance relevant for class separation.

The model was built with three latent variables (LVs) (*A* = 3) and mean centering as the pretreatment method (method = ‘centre’). Centering was selected instead of autoscaling or Pareto scaling to preserve the relative signal amplitudes while simplifying the interpretation of Variable Importance in Projection (VIP) scores and target-projection loadings (tpLoadings). Both the VIP and tpLoading profiles were used to identify the spectral bins most relevant to the classification, enabling a focused selection of peaks for subsequent quantitative integration. The class labels were encoded as +1 (one species) and −1 (the other), as required by the LDA routine. Model accuracy, sensitivity, specificity, and the area under the ROC curve (AUC) were computed to assess the discriminant performance.

In the PLS-LDA score plots, the continuous line represents the linear discriminant boundary in the latent-variable space. This boundary is defined by the linear discriminant function $c_{1}\cdot LV1+c_{2}\cdot LV2+c_{0}=0$, where the coefficients *c* are obtained by fitting a linear discriminant model to the PLS scores. Samples lying on one side of this line are assigned to *A. lixula*, whereas samples on the opposite side are assigned to *P. lividus* according to the sign of the discriminant function.

To identify the spectral regions most relevant for discriminating between *Arbacia lixula* and *Paracentrotus lividus*, a Subwindow Permutation Analysis (SPA) was applied to the integrated HR-MAS ^1^H-NMR data matrix [23,24]. SPA is a supervised Monte-Carlo–based variable-selection algorithm that repeatedly builds Partial Least Squares–Linear Discriminant Analysis (PLS-LDA) sub-models using randomly selected subsets of samples and variables. The method evaluates the statistical contribution of each variable by comparing prediction errors from the original and permuted datasets, providing a *p*-value and the corresponding Conditional Synergistic Score (COSS = −log_10_ *p*) as a quantitative index of variable importance. In this context, SPA refers exclusively to Subwindow Permutation Analysis, which ranks spectral variables according to their contribution to the predictive performance of the model by iteratively permuting local spectral windows. The most informative variables identified by SPA were compared with those highlighted by VIP and tpLoadings, and the overlap between these approaches was used to define robust sets of discriminant HR-MAS spectral regions for subsequent biological interpretation.

The analysis was performed in MATLAB using the original SPA implementation with the following parameters: Q = 15 (number of variables sampled per Monte-Carlo sub-model), K = 3 (folds for cross-validation), ratio = 0.7 (fraction of calibration samples), and N = 1000 (number of Monte-Carlo iterations). Pareto scaling was selected as pretreatment (“method = pareto”), as it balances normalization of variance while preserving metabolite intensity patterns. The maximum number of latent variables (A) was optimized automatically within each sub-model. Variables yielding COSS values greater than 2 (*p* < 0.01) were considered statistically significant contributors to class separation.

### 2.6. Pathway Enrichment and Topology Analysis

To place the species-specific metabolite profiles into a biochemical context, we performed pathway analysis using MetaboAnalyst v. 6.0 (Pathway Analysis module; www.metaboanalyst.ca, accessed on 1 December 2025). Two metabolite sets were compiled from the HR-MAS NMR results: one containing metabolites enriched in *Arbacia lixula* and another containing metabolites enriched in *Paracentrotus lividus*. Only metabolites that were unambiguously assigned in the HR-MAS spectra (Supplementary Table S1) and showed clear between-species differences according to the multivariate (PLS-LDA, SPA/COSS) and univariate analyses (Tables S2 and S3) were included. Metabolite names were matched to KEGG identifiers using the built-in compound name mapping tools.

For each species, pathway analysis was carried out in “compound list” mode using the KEGG metabolic pathway library. Enrichment was assessed by over-representation analysis based on the hypergeometric test, and pathway topology analysis was performed using relative betweenness centrality to compute a pathway impact score for each pathway. For every pathway, we report the total number of compounds in the pathway, the expected and observed number of hits, the nominal p-value, its –log10 transformation, Holm-adjusted p-value, false discovery rate (FDR) and impact value (Tables S1 and S2). The overall results are visualized as bubble plots of −log10(p) versus pathway impact for *A. lixula* and *P. lividus*, respectively. 

### 2.7. Ethical Considerations

The sea urchins were collected under the permission granted by the Dirección General de Pesca of the Generalitat Valenciana on 26 September 2024. All procedures involving animal collection and handling were conducted in compliance with institutional ethical guidelines approved by the University of Alicante (Exp. UA-2024-10-24_2). The study adhered to national and international regulations for the use of marine invertebrates in scientific research.

## 3. Results

### 3.1. HR-MAS Spectral Analysis

This study represents the first application of high-resolution magic angle spinning nuclear magnetic resonance (HR-MAS NMR) spectroscopy to the gonadal tissues of Mediterranean sea urchins. Representative ^1^H HR-MAS spectra obtained from *Arbacia lixula* and *Paracentrotus lividus* are shown in Supplementary Figure S1. A wide range of metabolites was identified using combined one-dimensional (1D) and two-dimensional (2D) HR-MAS experiments, including COSY, TOCSY, and HSQC, acquired on the same gonadal tissues. Spectral assignments (Supplementary Table S1) were based on direct signal identification, scalar correlations, heteronuclear couplings, and comparison with reference spectra and literature databases (HMDB; https://hmdb.ca/, accessed on 31 October 2025) [17,18,25,26,27]. The HR-MAS approach preserved the native biochemical composition of intact gonadal tissue without prior extraction or derivatization, enabling simultaneous detection of hydrophilic and hydrophobic compounds. 

Distinct spectral patterns were observed between *A. lixula* and *P. lividus*, involving both the presence/absence of specific resonances and variation in signal intensity (Supplementary Figure S1). In *A. lixula*, the spectrum was dominated by resonances of osmolytes and methylated amines (e.g., betaine, taurine, sarcosine, trimethylamine (TMA), and trimethylamine-N-oxide (TMAO)), as well as creatine and carnitine. In contrast, *P. lividus* spectra displayed higher intensities for amino acids (glycine, lysine, glutamine, glutamate, and proline) and stronger aliphatic signals from lipid methyl and methylene groups. These spectral trends suggested pronounced species-level differences in the chemical composition of gonadal tissues.

### 3.2. PLS-LDA Model

**Figure 1:**
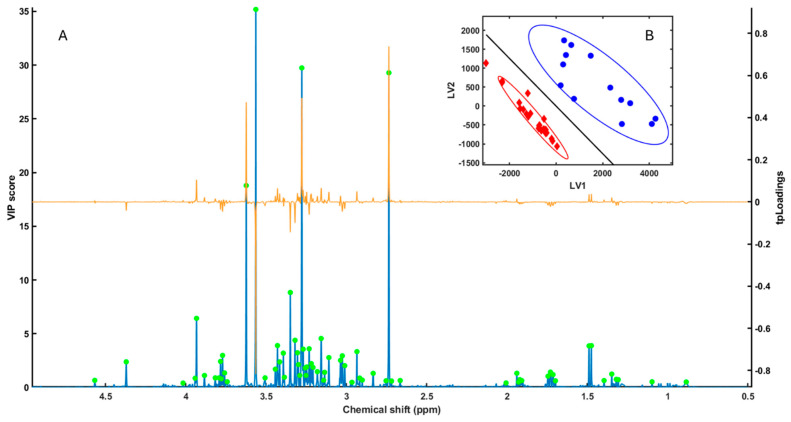
(**A**) VIP (blue line) and tpLoadings (orange line) values obtained from the PLS-LDA analysis of HR-MAS NMR spectra from *Arbacia lixula* and *Paracentrotus lividus* samples. Green dots indicate the spectral peaks with the highest VIP values, which were subsequently selected for the SPA (Subwindow Permutation Analysis) to further rank and select discriminant regions. (**B**) PLS-LDA score plot (LV1 vs. LV2) showing sample clustering according to species: blue circles correspond to *A. lixula* and red diamonds to *P. lividus*. The continuous line corresponds to the linear discriminant boundary in the PLS-LDA model; samples on each side of the line are assigned to one species or the other according to the sign of the discriminant function. Ellipses indicate the 95% confidence regions for each species cluster, illustrating within-group dispersion.

To identify the metabolites contributing most to the interspecific discrimination, a Partial Least Squares–Linear Discriminant Analysis (PLS-LDA) model was constructed using three latent variables Figure 1. The cumulative R^2^X = 0.93 and R^2^Y = 0.86 indicated that the selected components captured the majority of class-related variance while minimizing spectral noise. The PLS-LDA model built on the HR-MAS spectral data provided a clear separation between the gonadal metabolic fingerprints of *A. lixula* and *P. lividus* (Figure 1B). The model achieved perfect cross-validated classification performance, with zero misclassification error (error = 0), sensitivity and specificity equal to 1.00 for both species, and an area under the ROC curve (AUC) of 1.00. In the LV1 vs. LV2 score plot (Figure 1B), samples cluster according to species (the confidence ellipses outline the 95% dispersion regions for each species cluster), and the continuous line corresponds to the linear discriminant boundary of the PLS-LDA model. Geometrically, this boundary represents the set of scores for which the discriminant function equals zero and the posterior probabilities of class membership are equal; samples on one side of the line are classified as *A. lixula*, whereas samples on the other side are classified as *P. lividus*. 

Cross-validation and permutation testing confirmed the robustness of the classification model (AUC = 1), demonstrating strong predictive performance and reliable species-level separation. The Variable Importance in Projection (VIP) and target-projection loading (tpLoading) profiles highlighted consistent spectral regions with the highest discriminatory power (VIP > 1.0). These corresponded to signals associated with osmolytes, amino acids, and methylated metabolites (Figure 1A) [18].

### 3.3. SPA Variable Selection

To further validate the discriminant variables and quantify their statistical significance, a Subwindow Permutation Analysis (SPA) was applied to the integrated HR-MAS dataset. SPA repeatedly constructed PLS-LDA sub-models using randomly selected subsets of samples and variables to assess each variable’s contribution to class separation [24].

**Figure 2:**
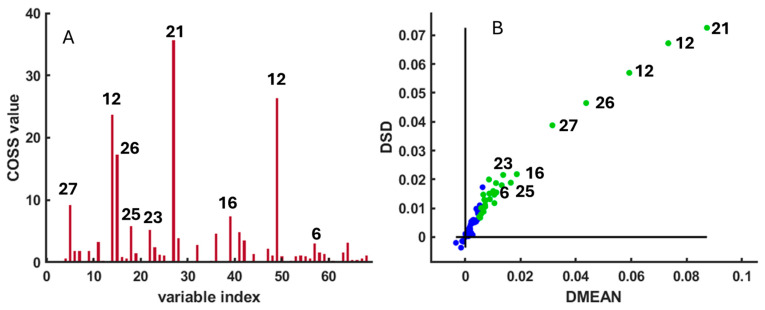
(**A**) COSS (Conditional Synergistic Score) values used to identify the most relevant spectral peaks differentiating *Arbacia lixula* and *Paracentrotus lividus* samples. (**B**) DMEAN versus DSD scatter plot. Variables with the highest discriminatory power are highlighted as green circles, whereas less relevant variables are shown as blue circles. The numbers appearing in the figures correspond to the compounds assigned in Supplementary Table S1.

**Figure 3:**
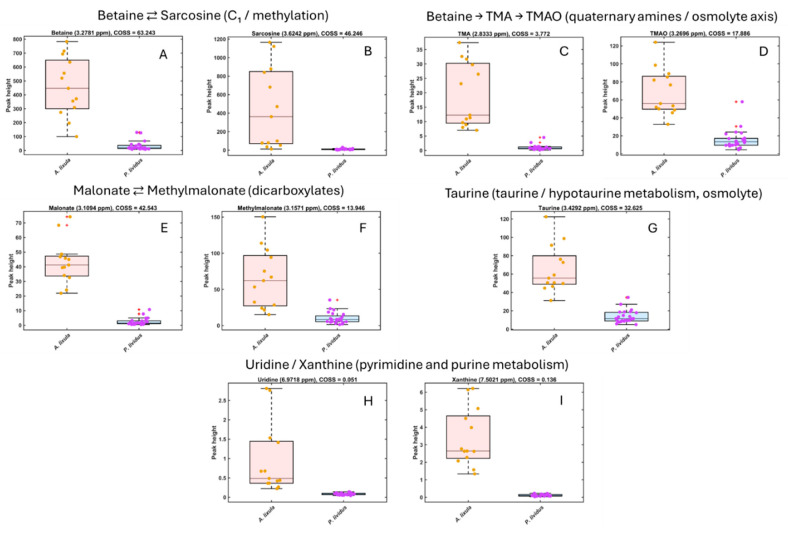
Boxplots of normalized HR-MAS ^1^H NMR peak intensities for key metabolites enriched in *Arbacia lixula* gonads. Metabolites are grouped according to known metabolic relationships. (**A**,**B**) Betaine and sarcosine illustrate the C_1_/methylation axis (betaine ⇄ sarcosine). (**C**,**D**) Betaine, trimethylamine (TMA) and trimethylamine N-oxide (TMAO) represent the quaternary-amine osmolyte/microbiota axis (betaine → TMA → TMAO). (**E**,**F**) Malonate and methylmalonate belong to the dicarboxylate/propionate-related pool (malonate ⇄ methylmalonate). (**G**) Taurine is a major osmolyte in the taurine/hypotaurine metabolism. (**H**,**I**) Uridine and xanthine are markers of pyrimidine and purine metabolism, respectively. Boxplots show the distribution of signal intensities for *A. lixula* (orange) and *P. lividus* (purple); dots represent individual urchin samples.

**Figure 4:**
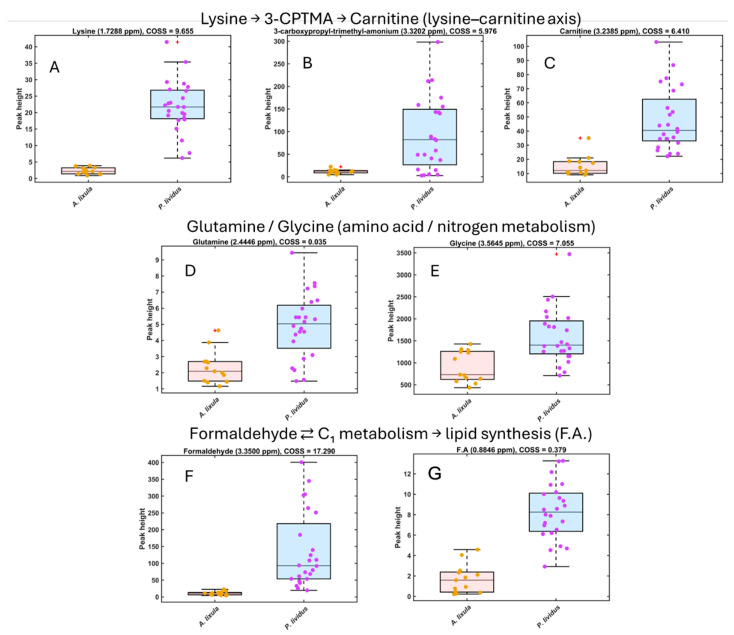
Boxplots of normalized HR-MAS ^1^H NMR peak intensities for key metabolites enriched in *Paracentrotus lividus* gonads. Metabolites are grouped according to known metabolic relationships. (**A–C**) Lysine, 3-carboxypropyl-trimethylammonium (3-CPTMA) and carnitine illustrate the lysine–carnitine axis (lysine → 3-CPTMA → carnitine). (**D**,**E**) Glutamine and glycine are representative amino acids within nitrogen and amino-acid metabolism. (**F**,**G**) Formaldehyde and the fatty acid (F.A.) signal reflect one-carbon intermediates feeding into energy and lipid metabolism (formaldehyde ⇄ C_1_ metabolism → lipid synthesis). Boxplots show the distribution of signal intensities for *A. lixula* (orange) and *P. lividus* (purple); dots represent individual urchin samples.

The analysis was performed using 1000 Monte Carlo iterations (N = 1000), Q = 15 variables per sub-model, and K = 3 folds for cross-validation, with 70% of samples used for model calibration (ratio = 0.7). Variables with the highest Conditional Synergistic Score (COSS) values (*p* < 0.01) were considered statistically significant discriminants and were highlighted as green circles in Figure 2A. The results of the SPA confirmed that several metabolites contributed most strongly to species differentiation, including betaine, sarcosine, glycine, malonate, taurine, formaldehyde, and lysine (Figure 2B). These variables were subsequently selected for quantitative comparison through spectral integration and visualized using boxplots of signal intensities (Figure 3 and Figure 4). The parameter choices and statistical procedure followed the original implementation of SPA described by [23].

### 3.4. Selected Metabolites and Boxplots

The metabolites showing the highest COSS values in the SPA were integrated to quantify species-specific differences (Figure 3 and Figure 4). Boxplots of normalized peak intensities confirmed statistically significant (*p* < 0.01) differences between *A. lixula* and *P. lividus* for all selected compounds.

In *A. lixula*, higher intensities were observed for osmolytes and methylated amines (betaine, sarcosine, TMA, TMAO), organic acids (malonate and methylmalonate), and energy-buffer molecules (carnitine and creatine). Additionally, nucleosides (uridine) and purine derivatives (xanthine) were detected exclusively or at higher levels in *A. lixula* (Figure 3).

Conversely, *P. lividus* samples showed elevated signals for amino acids (glycine and lysine) and for compounds likely linked to algal-derived or microbial methylotrophy, including formaldehyde, methanol, and 3-carboxypropyl-trimethyl-ammonium (3-CPTMA). These compounds were absent or near the detection limit in *A. lixula* (Figure 4).

In total, the analysis identified a consistent set of metabolites whose concentration differences account for the strong separation between the two sea urchin species. The full list of metabolites and their chemical shifts is provided in Supplementary Table S1, while Figure 2, Figure 3 and Figure 4 summarize their statistical and graphical representation. Together, these results confirm substantial biochemical divergence in the gonadal metabolomes of *A. lixula* and *P. lividus*, providing the basis for the mechanistic interpretations developed in the Discussion.

### 3.5. Pathway Analysis of Discriminatory Metabolites

To place the species-specific metabolite fingerprints into a pathway context, the sets of metabolites enriched in *A. lixula* and *P. lividus* were analyzed separately using the Pathway Analysis module of MetaboAnalyst (KEGG library, compound list mode). For each species, enrichment and pathway impact were evaluated by over-representation analysis combined with pathway topology metrics.

For *A. lixula*, the top-ranking pathway was “Glycine, serine and threonine metabolism”, with three matched metabolites out of 34 compounds in the pathway (raw *p* = 0.0014; FDR = 0.106; impact = 0.14). This reflects the clustering of betaine, sarcosine and glycine within this network. “One carbon pool by folate” also appeared over-represented (two hits out of 26; raw *p* = 0.0138; FDR = 0.52; impact = 0.14), linking these metabolites to folate-dependent one-carbon (C1) transfer reactions. “Taurine and hypotaurine metabolism” showed a trend towards enrichment (one hit out of 8; raw *p* = 0.0565; FDR = 1.0; impact = 0.43), consistent with the prominent taurine signal in *A. lixula* spectra (Figure 1). Additional pathways such as lysine degradation, arginine and proline metabolism, valine/leucine/isoleucine degradation, and purine and pyrimidine metabolism were detected with higher *p*-values and lower impact, but they are compatible with the observed accumulation of creatine, malonate/methylmalonate, uridine and xanthine in *A. lixula* gonads (Table S1).

For *P. lividus*, pathway analysis revealed a distinct pattern centred on nitrogen handling and amino-acid metabolism. “Glyoxylate and dicarboxylate metabolism” emerged as the top pathway, with three hits out of 32 compounds (raw *p* = 1.5 × 10^−4^; FDR = 0.0073; impact = 0.08), followed by “Nitrogen metabolism” (two hits out of 6; raw *p* = 1.9 × 10^−4^; FDR = 0.0073). Several additional nitrogen-related pathways were also over-represented, including “Arginine biosynthesis”, “Alanine, aspartate and glutamate metabolism” and “Glutathione metabolism” (two hits each; raw *p* < 0.005; FDR ≤ 0.06), the latter showing a relatively high pathway impact (0.44). Lysine degradation and porphyrin metabolism showed similar enrichment statistics, whereas one-carbon pool by folate, glycine/serine/threonine metabolism and purine/pyrimidine metabolism appeared with weaker statistical support but are consistent with the presence of methanol, formaldehyde and nucleotide derivatives in *P. lividus* gonads (Table S2).

**Figure 5:**
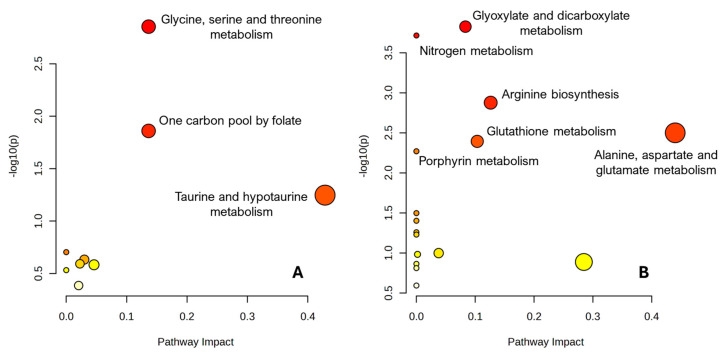
Pathway topology analysis of HR-MAS NMR metabolite sets using MetaboAnalyst. Each bubble represents a KEGG metabolic pathway, plotted according to its pathway impact (x-axis) and the statistical significance of the enrichment (−log10 of the raw *p*-value, y-axis). Bubble size is proportional to the number of matched metabolites in each pathway and bubble colour reflects the *p*-value (from yellow, less significant, to red, more significant). (**A**) Pathways associated with metabolites enriched in *Arbacia lixula*. (**B**) Pathways associated with metabolites enriched in *Paracentrotus lividus*. Full pathway names, hit numbers and statistics are provided in the Supplementary Tables S1 and S2.

These results are summarized in the MetaboAnalyst bubble plots shown in Figure 5. Each bubble represents a KEGG pathway, plotted according to pathway impact on the x-axis and the significance of the enrichment (−log10 of the raw *p*-value) on the y-axis; bubble size reflects the number of matched metabolites and bubble colour encodes the *p*-value. In the *A. lixula* plot (Figure 5A), the most prominent bubbles correspond to glycine/serine–threonine metabolism and the one-carbon pool by folate, with smaller but consistent bubbles for taurine/hypotaurine and nucleotide pathways. In contrast, the *P. lividus* plot (Figure 5B) is dominated by pathways related to glyoxylate/dicarboxylate metabolism, nitrogen metabolism and amino-acid/redox pathways, in line with the strong contribution of lysine, glycine, glutamine and glutathione-linked metabolites to the *P. lividus* profile. Representative HR-MAS spectral regions and boxplots for the most discriminatory SPA/COSS peaks are shown in Figures S1 (*A. lixula*-enriched metabolites) and S2 (*P. lividus*-enriched metabolites), where panel titles also report the corresponding COSS values.

## 4. Discussion

### 4.1. Core Metabolic Contrasts

Our comparative HR-MAS NMR metabolomic profiling of *Arbacia lixula* and *Paracentrotus lividus* reveals marked divergences in the biochemical composition of their gonadal tissues. These differences are not random but instead reflect species-specific physiological strategies shaped by ecology, diet, and evolutionary history. The metabolic contrasts can be organized into three main axes: (i) osmolyte-based cellular homeostasis, (ii) amino-acid and nitrogen-metabolism investment, and (iii) specific pathway activations linked to redox and immune balance.

In *A. lixula*, metabolites such as betaine (N-trimethylglycine), sarcosine (N-methylglycine), and taurine appear in markedly higher abundance than in *P. lividus*. These compounds are classic compatible osmolytes, maintaining intracellular osmotic equilibrium, stabilizing proteins, and protecting cellular structures against fluctuations in salinity, temperature, or oxidative stress. Betaine and taurine are dominant osmolytes in many marine invertebrates, where they help preserve enzyme functionality and membrane integrity under hyperosmotic or thermal stress [28,29]. The accumulation of these solutes in *A. lixula* thus likely represents an adaptive strategy toward homeostasis and resilience in environments characterized by greater variability in temperature and food availability.

Sarcosine, an intermediate in betaine and glycine metabolism, further indicates an active methyl-group turnover and one-carbon metabolic cycle. This enhanced methylation capacity might be related to the high metabolic flexibility of *A. lixula*, which occupies a broader trophic niche and frequently grazes on protein-rich biofilms or sessile invertebrates [2]. Taurine, beyond its osmotic role, acts as an antioxidant, cytoprotective and membrane-stabilizing molecule [30]. The combined accumulation of betaine, sarcosine, and taurine therefore suggests a metabolic orientation favouring stress tolerance and rapid cellular recovery—traits that align with *A. lixula*’s success in warm, oligotrophic, or “barren” Mediterranean habitats.

In contrast, *P. lividus* exhibited higher levels of amino acids such as glycine, glutamate, glutamine, arginine, proline, valine, and especially lysine, revealing a pronounced anabolic metabolism. These amino acids are central to nitrogen transport, protein synthesis, and energy storage and are often mobilized during gonadal maturation [3,4,31]. Elevated amino-acid pools have been associated with reproductive investment in echinoids, reflecting their role as precursors for gametogenic proteins and nucleotides. This pattern is consistent with previous biochemical analyses showing that *P. lividus* gonads exhibit a high proportion of free amino acids and lipids during active gametogenesis [8,32].

Among these, lysine deserves special attention due to its metabolic and developmental significance. Lysine is an essential amino acid that participates in multiple biosynthetic pathways, including the saccharopine pathway and carnitine biosynthesis, linking protein turnover to mitochondrial β-oxidation [33]. High lysine content in *P. lividus* may indicate both active anabolic processes and a readiness to synthesize carnitine for fatty-acid transport. This connection between lysine and lipid metabolism supports the interpretation of a reproductive-energy-storage strategy, where resources are channelled into gametogenesis and subsequent embryonic development. Historical studies already described elevated lysine levels during early developmental stages of sea urchins [31], supporting the view that this amino acid plays a crucial role in reproductive metabolism and growth.

Overall, *P. lividus* seems to prioritize anabolic efficiency—mobilizing amino acids and lipids as energy reserves—whereas *A. lixula* invests more in osmotic and antioxidant stability. These contrasting trends align with their ecological niches: *P. lividus* as a macroalgae-feeding species with seasonal reproductive peaks, and *A. lixula* as a thermotolerant omnivore sustaining reproduction even in nutrient-poor conditions [3,32].

Beyond the major amino acids and osmolytes, the presence of kynurenine in both species adds another layer of metabolic differentiation. Kynurenine is a central intermediate in the tryptophan-catabolic (kynurenine) pathway, which plays critical roles in redox regulation, immune modulation, and stress signalling. Recent studies have demonstrated the conservation of this pathway in a broad range of invertebrates, including molluscs, crustaceans, and annelids [34,35,36]. The detection of kynurenine in both *A. lixula* and *P. lividus* suggests that the pathway is also active in echinoderms, potentially contributing to immune regulation in gonadal tissues.

Kynurenine derivatives such as kynurenic acid and 3-hydroxykynurenine are known to balance oxidative processes and neuroactive functions, providing a biochemical interface between metabolism and cellular defence [37]. Its higher relative abundance in *A. lixula* may indicate a stronger oxidative-stress response, consistent with its osmolyte-enriched, stress-resilient phenotype. Moreover, marine natural products have been shown to modulate enzymes within this pathway [38], implying that environmental exposures or diet could influence its activity.

By contrast, *P. lividus*, with its lysine- and lipid-enriched anabolic metabolism, might rely less on kynurenine-mediated stress buffering and more on biosynthetic and energy pathways supporting reproductive output. This trade-off between stress-resilience (*A. lixula*) and anabolic efficiency (*P. lividus*) appears to define their respective metabolic strategies and may underpin their ecological partitioning within overlapping Mediterranean habitats.

Taken together, the pathway analysis complements the multivariate and univariate results by showing that the species-specific metabolite sets occupy different regions of metabolic pathway space. *A. lixula* sits at the centre of an osmolyte–C1–nucleotide network dominated by glycine/serine–threonine metabolism, the folate-linked one-carbon pool and taurine/hypotaurine metabolism, whereas *P. lividus* is anchored in nitrogen and amino-acid pathways, including glyoxylate/dicarboxylate metabolism, nitrogen metabolism and glutathione-coupled redox processes. This dual pattern is consistent with our proposed framework in which *A. lixula* adopts a resilience-oriented osmolyte strategy, while *P. lividus* follows an efficiency-oriented anabolic strategy, each optimizing biochemical resources in line with its feeding ecology, microhabitat and holobiont composition.

### 4.2. Secondary Metabolites and Microbial Mediation

Beyond the primary osmolytes and amino acids that define the core metabolic contrast between *A. lixula* and *P. lividus*, several additional metabolites provide deeper insight into their divergent physiological strategies. These include methylated amines (TMA and TMAO), dicarboxylic acids (malonate and methylmalonate), energy-buffer molecules (carnitine and creatine), nucleosides and purine derivatives (uridine and xanthine), and a unique set of compounds restricted to *P. lividus* (formaldehyde, methanol, and 3-carboxypropyl-trimethyl-ammonium). The possible presence of the methylxanthine alkaloid theophylline or its derivatives in *P. lividus* also deserves consideration. Together, these metabolites reveal the interplay between host metabolism, diet, and microbial co-metabolism within the holobiont framework.

In *A. lixula*, elevated concentrations of trimethylamine (TMA) and trimethylamine N-oxide (TMAO) represent one of the most distinctive metabolic traits. TMAO is a well-known organic osmolyte that stabilizes proteins and counteracts the destabilizing effects of urea and hydrostatic pressure in marine organisms [30]. In fish and invertebrates, it also serves as a chemical indicator of tissue freshness because microbial catabolism of TMAO releases TMA during post-mortem decay [27]. In living organisms, however, high TMAO levels typically indicate osmotic and structural homeostasis.

In marine invertebrates, TMA is mainly derived from microbial oxidation or demethylation of quaternary ammonium compounds such as choline, carnitine, and betaine [39]. The concurrent detection of TMA and TMAO in *A. lixula* is therefore compatible with an active metabolic exchange between the host and its associated microbiota. Bacterial taxa capable of oxidizing TMA to TMAO or further demethylating it to formaldehyde and ammonium have been identified in several marine systems [39]. Consequently, the enrichment of these metabolites in *A. lixula* may signal a symbiotic metabolic loop involving microbial methylamine cycling and host osmolyte regulation, a hypothesis that remains to be directly tested through integrated metabolomic–microbiomic analyses.

Two other compounds, malonate and methylmalonate, were notably more abundant in *A. lixula*. Malonate is a structural analogue of succinate and a competitive inhibitor of succinate dehydrogenase, thereby modulating mitochondrial electron transport and tricarboxylic-acid (TCA) cycle activity [40]. The co-accumulation of malonate and methylmalonate suggests the activation of propionate and odd-chain fatty-acid catabolism, pathways that feed into the TCA cycle via succinyl-CoA. In mammals and marine invertebrates alike, elevated methylmalonate concentrations often mark enhanced anaplerotic flux through propionyl-CoA metabolism [41]. In some molluscs, labelled propionate incorporation has confirmed methylmalonate-dependent biosynthesis of secondary metabolites [42].

The concurrent enrichment of carnitine and creatine in *A. lixula* further supports the idea of a dynamic and flexible energy metabolism. Carnitine facilitates the transport of long-chain fatty acids into mitochondria for β-oxidation, while creatine acts as a phosphagen buffer, maintaining ATP supply in tissues with variable energy demand [43,44,45]. The presence of both compounds suggests that *A. lixula* maintains an energetically responsive system capable of shifting between lipid oxidation and phosphocreatine buffering, consistent with an organism adapted to intermittent feeding and variable environmental stress.

The higher abundance of uridine and xanthine in *A. lixula* also provides clues to its cellular physiology. Uridine, a pyrimidine nucleoside, is essential for RNA synthesis, membrane phospholipid metabolism, and glycosylation reactions, whereas xanthine arises from purine catabolism via xanthine oxidase. Elevated levels of these compounds may indicate increased RNA turnover and oxidative metabolism in gonadal tissues. In other marine invertebrates, purine catabolites such as hypoxanthine and xanthine accumulate under oxidative or nutritional stress, acting as indicators of heightened metabolic activity [46]. The prominence of xanthine in *A. lixula* thus aligns with its stress-tolerant metabolic profile and may also reflect microbial purine degradation within its coelomic fluid [47].

In *P. lividus*, several metabolites—formaldehyde, methanol and 3-carboxypropyl-trimethyl-ammonium—were detected at high levels but were absent or negligible in *A. lixula*. These compounds are unusual in animal metabolomes and are plausibly linked to microbial or dietary methylotrophic processes. Macroalgae, the primary food source of *P. lividus*, release methanol and methylamines during cell-wall degradation, particularly under light stress or senescence [46]. Methanol oxidation by associated microbiota yields formaldehyde as a transient intermediate, which can subsequently react with amino groups or enter the tetrahydrofolate-linked one-carbon (C1) metabolism pathway [48]. The detection of 3-carboxypropyl-trimethyl-ammonium, a quaternary ammonium compound structurally related to glycine betaine, further supports a role for methylated substrates in this network. Rather than representing toxic end products, formaldehyde and methanol may therefore be transient metabolites within microbial–host co-metabolism. The absence of these compounds in *A. lixula* strengthens the view that *P. lividus* harbours a distinct methylotrophic microbial community capable of processing algal-derived C1 compounds.

Although the canonical methylxanthine theophylline (1,3-dimethylxanthine) was not unequivocally identified in our spectra, its occurrence or that of structurally related derivatives in *P. lividus* remains plausible. Methylxanthines are secondary metabolites widespread among marine and terrestrial algae, where they play roles in chemical defence and allelopathy [49]. Herbivorous invertebrates grazing on these algae may ingest and partially metabolize such compounds, leading to the appearance of demethylated derivatives such as xanthine, which was indeed abundant in our dataset. Microbial communities associated with *P. lividus* could further oxidize theophylline to xanthine via xanthine oxidase or related pathways [50]. If confirmed, the occurrence of methylxanthine derivatives would suggest an additional level of xenobiotic or detoxification metabolism in *P. lividus*, possibly linked to its algal diet and microbiota activity.

Collectively, the presence of these secondary metabolites is compatible with a complex biochemical interplay between host metabolism, diet and microbial mediation in both sea urchin species. In *A. lixula*, the constellation of TMA/TMAO, malonate, methylmalonate, carnitine, creatine, uridine and xanthine reflects an osmolyte-reinforced, energetically versatile metabolism that could be tightly coupled with microbial co-metabolism of methylated amines. In *P. lividus*, by contrast, the accumulation of formaldehyde, methanol and 3-carboxypropyl-trimethyl-ammonium points to a methylotrophic metabolic network likely sustained by an algal diet and associated microbiota. While our HR-MAS data alone cannot resolve the structure or function of these microbial assemblages, they provide metabolomic signatures that, together with existing microbiome studies, motivate a holobiont-level interpretation of species-specific metabolic strategies.

An important consideration when interpreting these patterns is that the gonadal metabolome is not static but changes with sex, body size and gametogenic stage. Previous studies on *P. lividus* have documented pronounced seasonal shifts in gonad biochemical composition and yield, as well as differences between males and females along the reproductive cycle [3,4,5,6,21]. Our sampling strategy, focused on adult individuals of both species collected at a single locality and during a restricted spring period, was designed to minimize gross environmental heterogeneity while capturing species-level contrasts. Nevertheless, some fraction of the within-species dispersion observed in Figure 2, Figure 3 and Figure 4 likely reflects unresolved variation in sex, individual size and precise reproductive status. The fact that *A. lixula* and *P. lividus* remain clearly separated in multivariate space despite this background variability indicates that species-level adaptations dominate the observed metabolomic divergence. Future HR-MAS studies explicitly stratified by sex, test diameter and gametogenic stage, and replicated across seasons, will be essential to partition intra- from inter-specific sources of variability and to refine the ecological interpretation of these metabolic fingerprints.

### 4.3. Integrative Ecological and Evolutionary Context

Although *Arbacia lixula* and *Paracentrotus lividus* share the same shallow rocky habitats and were sampled under identical environmental conditions, their gonadal metabolomes exhibit profound biochemical divergence. This divergence transcends simple interspecific variation, reflecting two distinct metabolic and ecological strategies shaped by diet, symbiotic microbiota, and evolutionary history. The data presented here support a dual adaptive framework: *A. lixula* adopts a resilience-oriented osmolyte strategy, whereas *P. lividus* follows an efficiency-oriented anabolic strategy.

A primary axis of metabolic differentiation between the two echinoids lies in their feeding ecology and nutrient assimilation. *P. lividus* is a strict herbivore that primarily consumes fleshy macroalgae rich in carbohydrates and nitrogen-poor biomass [3,21,51]. This diet promotes carbohydrate and amino-acid-oriented metabolism, reflected in the elevated levels of lysine, glycine, glutamine, and other amino acids, as well as higher lipid content in its gonads. These metabolites function as energy and structural reserves that sustain gametogenesis during seasonal reproductive peaks [3,32]. The observed anabolic profile of *P. lividus* aligns with its seasonally synchronized reproductive strategy, which depends on the availability of algal resources during cooler months [6].

Conversely, *A. lixula* exhibits omnivorous feeding behaviour, consuming filamentous and crustose algae, sessile invertebrates, and detrital biofilms [2]. Such dietary flexibility supplies a richer amino- and nitrogen-based substrate pool, fostering the synthesis of osmolytes such as betaine, taurine, and sarcosine. These metabolites enhance osmotic stability, oxidative protection, and protein maintenance, all of which are critical for surviving in nutrient-depleted, thermally variable “barren” habitats [7,52]. This metabolic orientation complements *A. lixula*’s capacity to maintain gonadal development under food-limited conditions, in contrast to the more resource-dependent *P. lividus* [6].

Emerging evidence indicates that the microbiota plays a pivotal role in modulating sea urchin metabolism, influencing both energy fluxes and chemical defence. The recent study by Arranz et al. (2025) documented substantial differences in the gut and coelomic microbiota of *A. lixula* and *P. lividus* across Mediterranean locations, including species-specific enrichment of choline-/carnitine-degrading and formaldehyde-/methanol-utilizing taxa [53]. Although our study did not characterize the microbiome directly, these published patterns are consistent with the metabolite profiles described here, in which *A. lixula* shows elevated TMA/TMAO and related osmolytes, whereas *P. lividus* exhibits higher levels of formaldehyde, methanol and other C1-linked compounds. We therefore interpret our results as metabolomic signatures that complement and extend the holobiont perspective emerging from microbiome surveys, rather than as independent proof of specific microbial pathways.

This microbiota–metabolite concordance supports a plausible mechanistic link between microbial functional potential and host metabolic profiles, which should be explicitly tested in future integrative studies. The TMA/TMAO cycle in *A. lixula* suggests microbial mediation of quaternary amine metabolism that contributes to osmotic balance and stress tolerance, whereas the formaldehyde–methanol network in *P. lividus* points to algal-derived methylotrophy within its holobiont. Comparable relationships between microbial metabolism and host chemistry have been documented in other echinoderms, where gut bacteria contribute to amino acid biosynthesis, short-chain fatty acid production, and detoxification processes [54,55].

From an evolutionary standpoint, *A. lixula* and *P. lividus* belong to distinct phylogenetic lineages that diverged several million years ago, likely under differing selective pressures [56,57]. This evolutionary separation has produced divergent regulatory architectures for energy metabolism and stress response. *A. lixula*, as a warm-adapted species expanding in a “tropicalizing” Mediterranean Sea, displays traits associated with environmental plasticity—osmolyte regulation, antioxidant balance, and flexible energy metabolism. *P. lividus*, on the other hand, retains the biochemical hallmarks of a temperate species, emphasizing nutrient assimilation and reproductive efficiency within more stable habitats [8,15].

Such divergence exemplifies metabolic evolution as an adaptive continuum: one lineage optimized for stability and reproduction, the other for resilience and persistence under stress. Importantly, both strategies are ecologically successful within their niches.

At the pathway level, the *A. lixula* profile is consistent with an osmolyte- and C1-oriented homeostatic strategy. The metabolites that dominate the *A. lixula* spectra (betaine, taurine, sarcosine, TMA/TMAO, carnitine, creatine, malonate, methylmalonate, uridine and xanthine) mapped preferentially onto glycine/serine–threonine metabolism and the one-carbon pool by folate, with glycine/serine metabolism emerging as the top enriched pathway (three hits; raw *p* = 0.0014; FDR ≈ 0.10). Together with the trend towards taurine/hypotaurine metabolism and the detection of purine and pyrimidine pathways, these results reinforce the view that *A. lixula* relies on a tightly integrated network of osmolytes and C1-linked reactions. This network combines the betaine–sarcosine–glycine cascade, taurine-based osmoprotection, creatine-mediated energy buffering and nucleotide turnover, providing osmotic and redox resilience in warm, food-limited microhabitats (Figure 5A and Table S1).

In contrast, the *P. lividus* pathway profile points to a nitrogen-rich, amino-acid- and lipid-centred anabolic strategy. The metabolites enriched in *P. lividus* (lysine, glycine, glutamine, fatty acids, methanol, formaldehyde and 3-carboxypropyl-trimethylammonium) mapped strongly onto glyoxylate and dicarboxylate metabolism and nitrogen metabolism, both of which remained significant after FDR correction (three and two hits, respectively; FDR = 0.0073). Additional enrichment of arginine biosynthesis, alanine/aspartate/glutamate metabolism and glutathione metabolism indicates extensive interconversion among amino-acid families, active handling of inorganic and organic nitrogen, and a prominent role for glutathione-linked redox control. These pathways align with the accumulation of free amino acids and fatty acid signals in *P. lividus* gonads and support the idea that this species channels resources into amino-acid and lipid reserves that can fuel both protein biosynthesis and energy production via the TCA cycle (Figure 5B and Table S2).

A key limitation of this study is that we did not directly characterize the microbial communities associated with the gonadal tissues (e.g., via 16S rRNA amplicon sequencing or metagenomics). Our interpretations of host–microbiota interactions are therefore based solely on metabolite patterns and on previously published microbiome data for these species and related echinoids [53,54,55]. These links should be viewed as hypothesis-generating rather than demonstrative. Future work integrating HR-MAS metabolomics with parallel microbiome profiling will be essential to directly test the proposed functional connections between specific microbial taxa, methylated substrates and host metabolic strategies.

## 5. Conclusions

This study provides the first comparative analysis of the gonadal metabolomes of the Mediterranean sea urchins *Arbacia lixula* and *Paracentrotus lividus* using HR-MAS NMR spectroscopy. The results reveal clear species-specific metabolic differentiation that mirrors their ecological and physiological divergence.

Overall, *A. lixula* exhibits an osmolyte- and redox-oriented metabolic phenotype, characterized by elevated levels of betaine, taurine, sarcosine, TMAO, carnitine and creatine, consistent with enhanced cellular homeostasis and stress tolerance in warm, food-limited habitats. In contrast, *P. lividus* shows an amino-acid- and lipid-enriched anabolic profile dominated by lysine, glycine, glutamine and fatty acids, supporting a strategy centred on energy storage and reproductive investment. Additional metabolites linked to methylated and C1 compounds are compatible with microbial mediation and suggest functional connections between host metabolism, diet and holobiont microbiota.

By linking metabolomic fingerprints with ecological function and feeding behaviour, our work supports a conceptual framework in which *A. lixula* and *P. lividus* represent complementary adaptive solutions—one emphasizing resilience and homeostatic control, the other prioritizing anabolic efficiency. HR-MAS NMR metabolomics thus emerges as a powerful, hypothesis-generating approach to investigate adaptive biochemical diversity in marine invertebrates. Future studies integrating metabolomics, microbiomics and transcriptomics across seasons, sexes and environmental gradients will be essential to test these hypotheses and to understand how echinoid holobionts respond to ongoing ocean warming and acidification. 

## Data Availability

The data presented in this study are available on request from the corresponding author.

## Supplementary Materials

The following supporting information can be downloaded at https://www.mdpi.com/article/10.3390/metabo15120787/s1, Figure S1: Representative 1D 1H CPMG HR-MAS NMR spectrum of gonadal tissues of *Arbacia lixula* (orange line) and *Paracentrotus lividus* (purple line); Table S1: Assignment of 1D 1H CPMG HR-MAS NMR of gonadal tissues of *Arbacia lixula* and *Paracentrotus lividus*. The table lists the peak number used in Figure 1, the chemical shift (δ, ppm), the compound name, and the multiplicity observed in the spectra; Table S2: Pathway analysis of metabolites enriched in *Arbacia lixula* gonads using MetaboAnalyst. Metabolite set enrichment and pathway topology analysis were performed in MetaboAnalyst (version 6.0) using the KEGG pathway library and the “compound list” mode. The table reports, for each KEGG pathway, the total number of compounds in the pathway (Total), the expected number of hits under the null hypothesis (Expected), the number of matched metabolites from the *A. lixula* set (Hits), the nominal *p*-value for over-representation (Raw *p*), the corresponding −log10(*p*), the Holm-adjusted *p*-value, the false discovery rate (FDR) and the pathway impact score derived from topology analysis. Pathways are sorted by increasing Raw *p*-value; Table S3: Pathway analysis of metabolites enriched in *Paracentrotus lividus* gonads using MetaboAnalyst. Metabolite set enrichment and pathway topology analysis were performed in MetaboAnalyst (version 6.0) using the KEGG pathway library and the “compound list” mode. The table shows, for each KEGG pathway, the total number of compounds in the pathway (Total), the expected number of hits under the null hypothesis (Expected), the number of matched metabolites from the *P. lividus* set (Hits), the nominal *p*-value for over-representation (Raw *p*), the corresponding –log10(*p*), the Holm-adjusted *p*-value, the false discovery rate (FDR) and the pathway impact score derived from topology analysis. Pathways are sorted by increasing Raw *p*-value.
