# Supplementary material for: Osmolytes vs. Anabolic Reserves: Contrasting Gonadal Metabolomes in Two Sympatric Mediterranean Sea Urchins
Source: Metabolites. 2025 Dec 10;15(12):787. doi: 10.3390/metabo15120787 (PMC12735097; doi:10.3390/metabo15120787)
Supplement: Supplementary file 1 [file metabolites-15-00787-s001.zip › metabolites-3993758-supplementary.pdf]

*Article*

# Osmolytes vs. Anabolic Reserves: Contrasting Gonadal Metabolomes in Two Sympatric Mediterranean Sea Urchins

**Estela Carbonell-Garzón [1], Ricardo Ibanco-Cañete [1], Pablo Sanchez-Jerez [1], Frutos C. Marhuenda Egea [2,*]**

[1] Department of Marine Sciences and Applied Biology, University of Alicante. Carretera San Vicente del Raspeig s/n, 03690, Alicante, Spain; ricardo.ibanco@gmail.com (R. I-C.); estela.carbonell@ua.es (E.C.-G); pablo.sanchez@ua.es (P.S.-J.)

[2] Department of Biochemistry and Molecular Biology and Agricultural Chemistry and Edafology, University of Alicante. Carretera San Vicente del Raspeig s/n, 03690, Alicante, Spain; frutos@ua.es

* Correspondence: frutos@ua.es

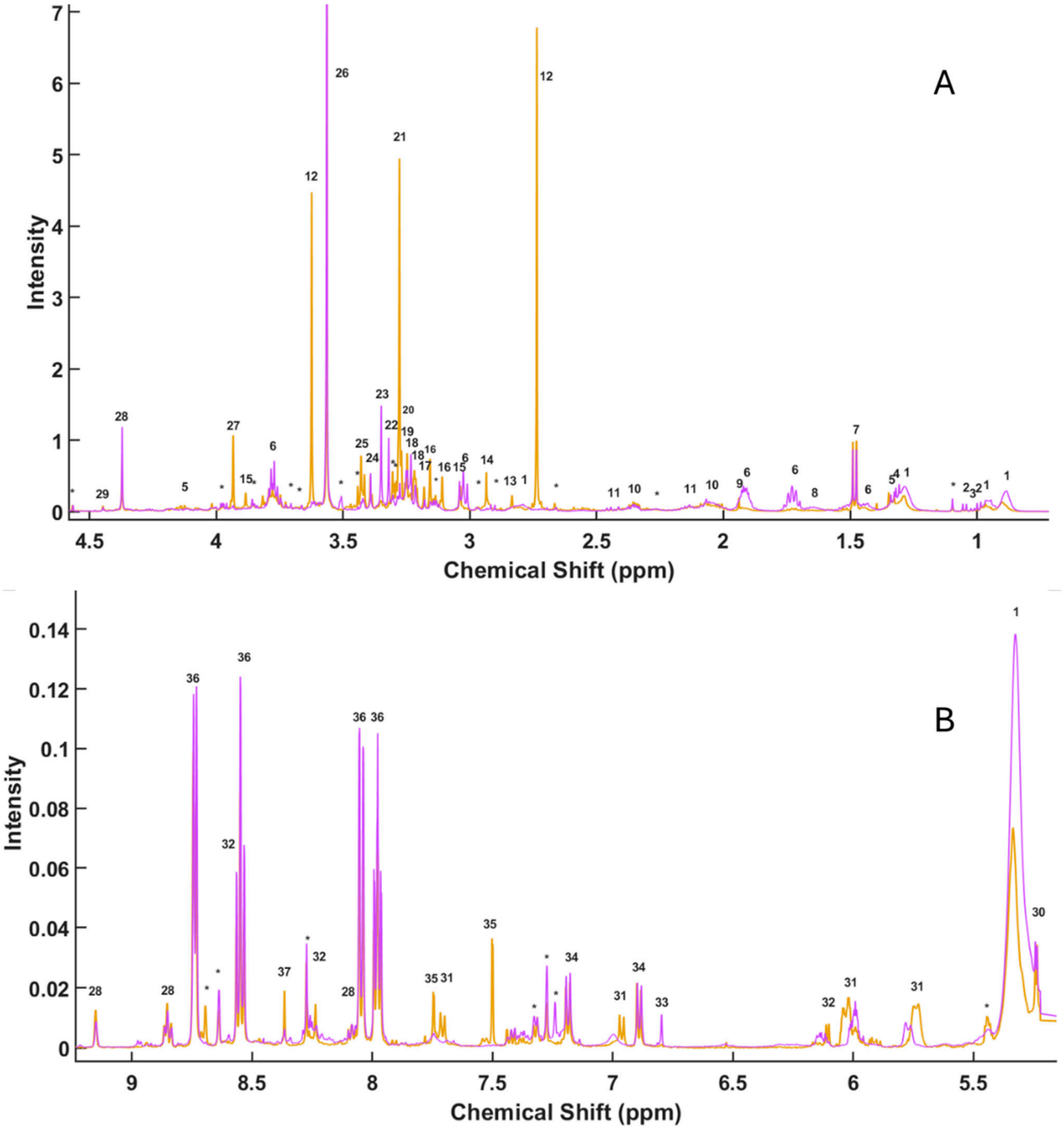


**Figure S1.** Representative 1D 1H CPMG HR-MAS NMR spectrum of gonadal tissues of *Arbacia lixula* (orange line) and *Paracentrotus lividus* (purple line).

**Table S1.** Assignment of 1D 1H CPMG HR-MAS NMR of gonadal tissues of *Arbacia lixula* and *Paracentrotus lividus*. The table lists the peak number used in Figure 1, the chemical shift (δ, ppm), the compound name, and the multiplicity observed in the spectra.

| Peak | δ (ppm) | Compound | Multiplicity |
|---|---|---|---|
| **1** | 0.8846 | F.A. | m |
| **1** | 0.9657 | F.A. | m |
| **2** | 0.9851 | Valine | d |
| **3** | 1.0101 | Isoleucine | d |
| **2** | 1.0416 | Valine | d |
| * | 1.0962 | Unassigned | s |
| **1** | 1.284 | F.A. | m |
| **4** | 1.3074 | Treonine | d |
| **5** | 1.3262 | Lactate | d |
| **6** | 1.4307 | Lysine | m |
| **7** | 1.4754 | Alanine | d |
| **8** | 1.6562 | Arginine | m |
| **6** | 1.7288 | Lysine | m |
| **6** | 1.9209 | Lysine | m |
| **9** | 1.9389 | Acetate | s |
| **10** | 2.068 | Glutamate | m |
| **11** | 2.1344 | Glutamine | m |
| * | 2.2653 | Unassigned | m |
| **10** | 2.3573 | Glutamate | m |
| **11** | 2.4446 | Glutamine | m |
| * | 2.6954 | Unassigned | s |
| **12** | 2.7357 | Sarcosine | s |
| **1** | 2.7893 | F.A. | m |
| **13** | 2.8333 | TMA | s |
| * | 2.9338 | Unassigned | d |
| **14** | 2.9349 | N-Methylhydantoin | s |
| * | 2.9741 | Unassigned | d |
| **6** | 3.0258 | Lysine | t |
| **15** | 3.0375 | Creatine | s |
| **16** | 3.1094 | Malonate | s |
| * | 3.1351 | Unassigned | d |
| **16** | 3.1556 | Malonate | s |
| **17** | 3.1571 | Methylmalonate | s |
| **18** | 3.2183 | Choline | s |
| **18** | 3.2326 | Choline | s |
| **19** | 3.2385 | Carnitine | s |
| **19** | 3.2476 | Carnitine | s |
| **20** | 3.2696 | TMAO | s |
| **21** | 3.2781 | Betaine | s |
| * | 3.3037 | Unassigned | s |
| * | 3.3048 | Unassigned | s |

| | | | |
|---|---|---|---|
| **22** | 3.3202 | 3-carboxypropyl-trimethyl-ammonium | s |
| **23** | 3.35 | Formaldehyde | s |
| **24** | 3.3918 | Methanol | s |
| **25** | 3.4292 | Taurine | t |
| * | 3.44 | Unassigned | s |
| * | 3.50763 | Unassigned | s |
| **26** | 3.56447 | Glycine | s |
| **12** | 3.6242 | Sarcosine | s |
| * | 3.6242 | Unassigned | s |
| * | 3.662 | Unassigned | s |
| **6** | 3.7717 | Lysine/Alanine | m |
| * | 3.8589 | Unassigned | m |
| **15** | 3.8846 | Creatine | s |
| **27** | 3.9341 | Betaine | s |
| * | 3.9741 | Unassigned | m |
| **5** | 4.1255 | Lactate | q |
| **28** | 4.3716 | Trigonelline | s |
| **29** | 4.446 | Inosine | s |
| **30** | 5.2421 | Glucose | d |
| **1** | 5.3261 | F.A. | s |
| * | 5.4335 | Unassigned | m |
| **31** | 5.7291 | Uridine | m |
| **31** | 6.0202 | Uridine | m |
| **32** | 6.1005 | IMP | d |
| **33** | 6.7965 | Thymol | s |
| **34** | 6.8985 | Tyrosine | d |
| **31** | 6.9718 | Uridine | d |
| **34** | 7.1783 | Tyrosine | d |
| * | 7.2399 | Unassigned | s |
| * | 7.2751 | Unassigned | s |
| * | 7.3286 | Unassigned | d |
| **35** | 7.5021 | Xanthine | s |
| **31** | 7.6993 | Uridine | d |
| **35** | 7.7477 | Xanthine | s |
| **36** | 7.978 | Kynurenine | t |
| **36** | 8.0532 | Kynurenine | d |
| **28** | 8.088 | Trigonelline | t |
| **32** | 8.2373 | IMP | s |
| * | 8.2736 | Unassigned | s |
| **37** | 8.3667 | Adenosine | s |
| **36** | 8.5501 | Kynurenine | t |
| **32** | 8.6014 | IMP | s |
| * | 8.6381 | Unassigned | s |
| * | 8.6953 | Unassigned | s |
| **36** | 8.7312 | Kynurenine | d |

| | | | |
|---|---|---|---|
| **28** | 8.8508 | Trigonelline | t |
| **28** | 9.1522 | Trigonelline | s |

Abbreviations: s, singlet; d, doublet; t, triplet; q, quartet; m, multiplet.

**Table S2.** Pathway analysis of metabolites enriched in *Arbacia lixula* gonads using MetaboAnalyst. Metabolite set enrichment and pathway topology analysis were performed in MetaboAnalyst (version 6.0) using the KEGG pathway library and the "compound list" mode. The table reports, for each KEGG pathway, the total number of compounds in the pathway (Total), the expected number of hits under the null hypothesis (Expected), the number of matched metabolites from the *A. lixula* set (Hits), the nominal p-value for over-representation (Raw p), the corresponding –log10(p), the Holm-adjusted p-value, the false discovery rate (FDR) and the pathway impact score derived from topology analysis. Pathways are sorted by increasing Raw p-value.

| Pathway | Total | Expected | Hits | Raw p | -log10(p) | Holm adjust | FDR | Impact |
|---|---|---|---|---|---|---|---|---|
| **Glycine, serine and threonine metabolism** | 34 | 0.24578 | 3 | 0.0013989 | 2.8542 | 0.10632 | 0.10632 | 0.1364 |
| **One carbon pool by folate** | 26 | 0.18795 | 2 | 0.013803 | 1.86 | 1 | 0.5245 | 0.13637 |
| **Taurine and hypotaurine metabolism** | 8 | 0.057831 | 1 | 0.056544 | 1.2476 | 1 | 1 | 0.42857 |
| **Lysine degradation** | 30 | 0.21687 | 1 | 0.19767 | 0.7041 | 1 | 1 | 0 |
| **Arginine and proline metabolism** | 36 | 0.26024 | 1 | 0.23275 | 0.6331 | 1 | 1 | 0.02996 |
| **Valine, leucine and isoleucine degradation** | 40 | 0.28916 | 1 | 0.25537 | 0.5928 | 1 | 1 | 0.02264 |
| **Pyrimidine metabolism** | 41 | 0.29639 | 1 | 0.26093 | 0.5835 | 1 | 1 | 0.04595 |
| **Fatty acid biosynthesis** | 47 | 0.33976 | 1 | 0.29353 | 0.5323 | 1 | 1 | 0 |
| **Purine metabolism** | 71 | 0.51325 | 1 | 0.41153 | 0.3856 | 1 | 1 | 0.02051 |

**Table S3.** Pathway analysis of metabolites enriched in *Paracentrotus lividus* gonads using MetaboAnalyst. Metabolite set enrichment and pathway topology analysis were performed in MetaboAnalyst (version 6.0) using the KEGG pathway library and the "compound list" mode. The table shows, for each KEGG pathway, the total number of compounds in the pathway (Total), the expected number of hits under the null hypothesis (Expected), the number of matched metabolites from the *P. lividus* set (Hits), the nominal p-value for over-representation (Raw p), the corresponding –log10(p), the Holm-adjusted p-value, the false discovery rate (FDR) and the pathway impact score derived from topology analysis. Pathways are sorted by increasing Raw p-value.

| Pathway | Total | Expected | Hits | Raw p | -log10(p) | Holm adjust | FDR | Impact |
|---|---|---|---|---|---|---|---|---|
| **Glyoxylate and dicarboxylate metabolism** | 32 | 0.12851 | 3 | 0.00014922 | 3.8262 | 0.011341 | 0.0073133 | 0.08333 |
| **Nitrogen metabolism** | 6 | 0.024096 | 2 | 0.00019246 | 3.7157 | 0.014434 | 0.0073133 | 0 |
| **Arginine biosynthesis** | 15 | 0.060241 | 2 | 0.0013277 | 2.8769 | 0.098253 | 0.033636 | 0.12626 |
| **Alanine, aspartate and glutamate metabolism** | 23 | 0.092369 | 2 | 0.003158 | 2.5006 | 0.23054 | 0.058285 | 0.43991 |
| **Glutathione metabolism** | 26 | 0.10442 | 2 | 0.004037 | 2.3939 | 0.29067 | 0.058285 | 0.10342 |
| **Lysine degradation** | 30 | 0.12048 | 2 | 0.0053684 | 2.2702 | 0.38115 | 0.058285 | 0 |
| **Porphyrin metabolism** | 30 | 0.12048 | 2 | 0.0053684 | 2.2702 | 0.38115 | 0.058285 | 0 |
| **Phosphonate and phosphinate metabolism** | 8 | 0.032129 | 1 | 0.031769 | 1.498 | 1 | 0.3018 | 0 |
| **Biotin metabolism** | 10 | 0.040161 | 1 | 0.039583 | 1.4025 | 1 | 0.33426 | 0 |
| **Histidine metabolism** | 14 | 0.056225 | 1 | 0.055061 | 1.2592 | 1 | 0.40694 | 0 |
| **Butanoate metabolism** | 15 | 0.060241 | 1 | 0.058899 | 1.2299 | 1 | 0.40694 | 0 |
| **One carbon pool by folate** | 26 | 0.10442 | 1 | 0.1003 | 0.9987 | 1 | 0.60795 | 0.03783 |
| **Lipoic acid metabolism** | 27 | 0.10843 | 1 | 0.10399 | 0.983 | 1 | 0.60795 | 0.00189 |
| **Glycine, serine and threonine metabolism** | 34 | 0.13655 | 1 | 0.12949 | 0.8878 | 1 | 0.69242 | 0.28464 |
| **Arginine and proline metabolism** | 36 | 0.14458 | 1 | 0.13666 | 0.8644 | 1 | 0.69242 | 0 |
| **Pyrimidine metabolism** | 41 | 0.16466 | 1 | 0.1544 | 0.8114 | 1 | 0.73338 | 0 |
| **Purine metabolism** | 71 | 0.28514 | 1 | 0.25478 | 0.5938 | 1 | 1 | 0 |
